# Sleep disturbances in late pregnancy: associations with induction of labor

**DOI:** 10.1007/s00404-024-07492-4

**Published:** 2024-04-05

**Authors:** Henna Lähde, Hasse Karlsson, Linnea Karlsson, Laura Perasto, Viliina Varis, Kirsi Rinne, E. Juulia Paavonen, Päivi Polo-Kantola

**Affiliations:** 1 Department of Obstetrics and Gynecology, Turku University Hospital and University of Turku, Savitehtaankatu 5, 20521 Turku, Finland; 2 FinnBrain Birth Cohort Study, Department of Clinical Medicine, Turku Brain and Mind Center, University of Turku, Turku, Finland; 3 Department of Psychiatry, Turku University Hospital and University of Turku, Turku, Finland; 4 Centre for Population Health Research, Turku University Hospital and University of Turku, Turku, Finland; 5 Department of Clinical Medicine, Pediatrics and Adolescent Medicine, Turku University Hospital and University of Turku, Turku, Finland; 6 The Department of Public Health and Welfare, Finnish Institute for Health and Welfare, Helsinki, Finland; 7 Child Psychiatry, University of Helsinki and Helsinki University Hospital, Helsinki, Finland; 8 Sleep Research Unit, University of Turku, Turku, Finland

**Keywords:** Labor induction, Mother, Pregnant, Sleep quality, Woman

## Abstract

**Purpose:**

Sleep disturbances, which are common during pregnancy, may compromise labor. Nevertheless, little is known about associations between sleep disturbances and the likelihood of ending up induction of labor (IOL). Accordingly, we aimed to evaluate the connections between sleep disturbances during pregnancy and IOL.

**Methods:**

Altogether 1778 women from the FinnBrain Birth Cohort Study with gestation weeks over 37 + 6 were enrolled in the study. The women were divided into IOL (n = 331) and spontaneous onset of labor (SOL, n = 1447) groups. Sleep disturbances in late pregnancy were evaluated using the Basic Nordic Sleep Questionnaire. Logistic regression analyses were conducted with adjustments for age, body mass index, parity, smoking, and depressive symptoms.

**Results:**

Sleep disturbances were frequent in both IOL and SOL groups. In the IOL group 43.0% and in the SOL group 39.0% had poor general sleep quality (*P* = 0.186). Nocturnal awakenings occurred most commonly, in 94.0% and 93.9%, respectively (*P* = 0.653). In the IOL group, more women (22.7%) were habitual snorers than in the SOL group (17.0%, *P* = 0.017), however, the difference lost the statistical significance in adjusted analysis (*P* = 0.848). Women in the IOL group were more likely to be short sleepers (< 7 h) compared to those in the SOL group (20.2% and 15.4%, respectively, *P* = 0.034) with no difference after adjustment (*P* = 0.133). The two groups showed no differences in sleep loss (*P* = 0.252).

**Conclusions:**

Deterioration in sleep quality was noticeable in pregnant women, but it was unconnected with IOL. As the frequency of IOL is increasing, more research for related risk factors is needed.

## What does this study add to the clinical work

**Table:** 

Sleep disturbances, especially insomnia symptoms, occurred frequently in late pregnancy. However, they were not associated with the likelihood of ending up to induction of labor.	

## Introduction

The rate of induction of labor (IOL) is increasing [1]. Nowadays, 25–30% of labors are induced [2, 3]. The rise in induction rates is partly explained by increased obesity and maternal age [1, 4] and by knowledge about IOL timing in complicated pregnancies [5]. Other important factors include women’s desire for IOL [6–8], maternal tiredness [6, 8, 9], and social reasons [10].

In pregnant women, sleep is commonly disrupted as a result of multiple factors that vary along pregnancy, such as hormonal and anatomical alterations, increased urinary frequency and physical discomfort [11]. Sleep disturbances are common during pregnancy and peak at the end of pregnancy [11–13]. In late pregnancy, insomnia symptoms, like difficulty to fall asleep and nocturnal awakenings increase and sleep duration decrease [12, 14, 15]. Additionally, sleep disordered breathing (SDB), especially snoring [16], as well as morning and daytime sleepiness [11, 12, 14] increase.

Sleep disturbances have shown to be associated with pregnancy complications [17–19]. For example, women with short or long sleep duration, or with SDB, have found to have more pre-eclampsia and gestational diabetes [17], complications which often may lead to IOL. In addition, women with advanced age are at higher risk for adverse obstetrical outcomes [20] and also in higher risk for sleep disturbances [21], which accordingly increase the risk for having IOL in those women. Sleep disturbances have been shown to be related to labor as well. Women with sleep disturbances experience more pain during delivery [22], have longer duration of labor [23], and deliver at a lower gestational age [24]. Short sleep duration, usually referred to sleep under 7 h per night [25], increases risk for preterm birth [18, 19] and cesarean section (CS) [23]. Furthermore, SDB have been shown to be associated with increased risk for preterm birth and CS [19].

Even though sleep disturbances during pregnancy have shown to be associated with pregnancy and delivery complications, little is known about direct associations with IOL. One of the mediators between sleep disturbances and IOL could be cytokines. For instance, there is a trend for overexpression of anti-inflammatory T helper 2 lymphocyte produced cytokines (Th2 cytokines) in early pregnancy and a shift towards a Th1 produced pro-inflammatory cytokine profile when pregnancy proceeds towards labor [26]. Poor sleep quality in late pregnancy has been related to increased pro-inflammatory interleukin-6 concentration, one of the Th1 cytokines [26]. Accordingly, sleep deprivation and poor sleep quality have shown to increase pro-inflammatory cytokine levels, and thus, it can be argued that sleep disturbances are stressors [26–28], which may also lead to IOL. However, according to a sole previous study with 35 pregnant women, no differences were found in sleep duration and nightly wake-up time between women with IOL and spontaneous onset of labor (SOL) [7]. In the present study, we aimed to evaluate associations between sleep disturbances and the likelihood to end up in IOL. We hypothesized that women suffering from sleep disturbances, especially from insomnia and sleepiness symptoms, SDB and sleep loss, are more likely to have IOL.

## Methods

Our study was a part of a larger population-based pregnancy cohort, the FinnBrain Birth Cohort Study, conducted at Turku University, Finland. In the cohort, families are followed during pregnancy and years thereafter (www.finnbrain.fi) [29]. The women were recruited from Turku and Åland hospital districts at their routine ultrasound appointment in early pregnancy between 2011 and 2015. All volunteers with adequate language skills to fill in the questionnaires were enrolled if they were willing to participate after providing oral and written information and written consent.

**Fig. 1:**
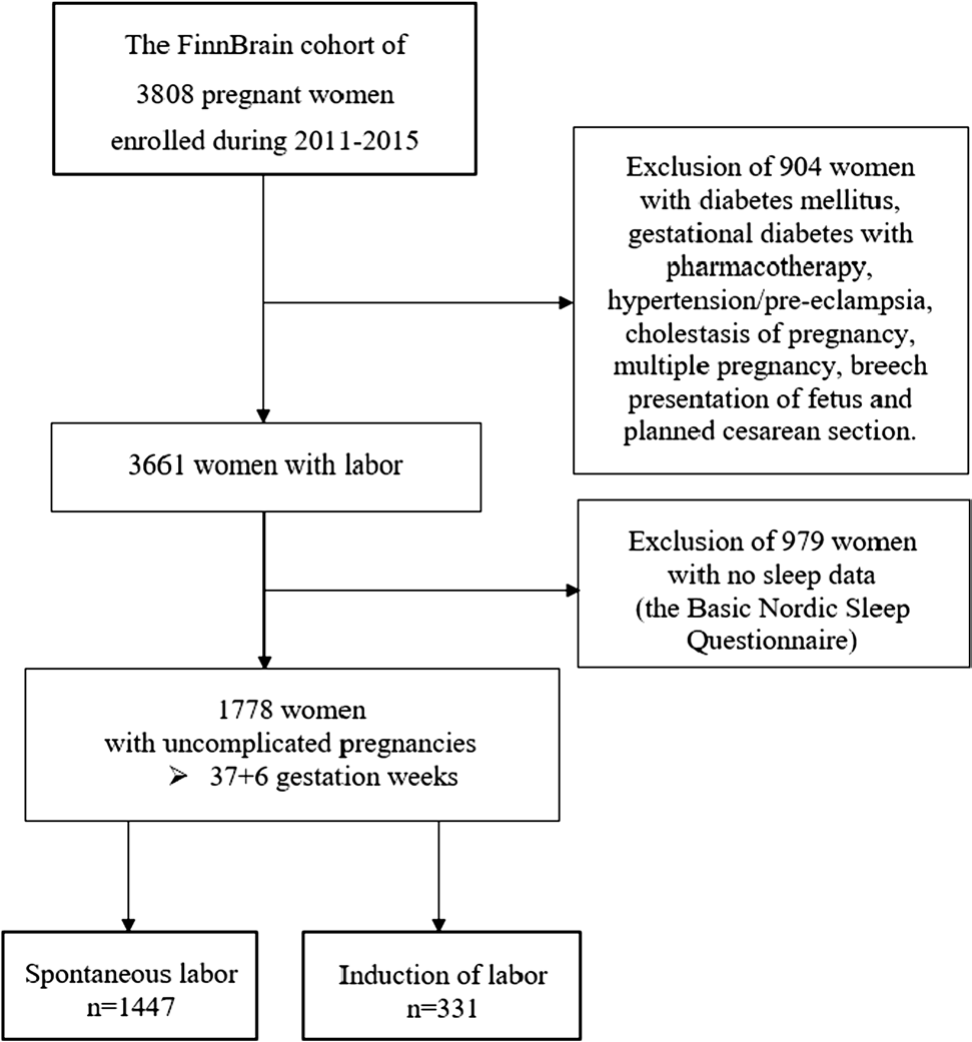
Flow chart of the study

The entire FinnBrain cohort consisted of 3808 women. As our primary aim was to evaluate the likelihood to end with IOL in full-term pregnancies, women with pregnancy complications which could lead to IOL (e.g. pregnancy hypertension/pre-eclampsia, pre-pregnancy diabetes mellitus, gestational diabetes with pharmacotherapy, cholestasis in pregnancy), as well as pregnancies with multiple fetuses, breech presentation fetuses, delivery < 38 gestation week (gwk), and planned CS were excluded. The researchers obtained IOL information from the Finnish Medical Birth Register and rechecked it from medical records. In the present study, the study population included all women (*n* = 1778) who answered the sleep questionnaire after delivery in the postpartum ward. Of them, the exact date of the response was available in 1502 women, of which 1497 had answered within seven days and five later (one in day 8, one in day 9, one in day 12, one in day 16 and one in day 30). In 276 women, the date of the reply was missing in the questionnaire. Two groups were formed: (1) IOL group with 331 women and (2) control (SOL) group with 1447 women. Figure 1 shows a flowchart of the study. Background information was drawn from the baseline questionnaire assessed around gwk 14, including age (years), body mass index (BMI; kg/m^2^), parity (primiparous/multiparous), smoking (yes/no), and depressive symptoms (Edinburgh Postnatal Depression Scale [EPDS]) [30] (Table 1).

**Table 1:** Basis characteristics

	All *n* = 1778	Spontaneous labor *n* = 1447	Induction of labor *n* = 331	
	Mean ± SD	Mean ± SD	Mean ± SD	*P-*value
Maternal age (years)*	30.1 ± 4.4	29.9 ± 4.4	30.7 ± 4.5	0.007
BMI (kg/m^2^)^*^	24.2 ± 4.4	23.8 ± 4.1	25.7 ± 5.4	< 0.001
EPDS*	5.2 ± 4.0	5.1 ± 4.0	5.3 ± 4.2	0.600
Gestational age*	40 + 2 ± 7	40 + 1 ± 7	40 + 4 ± 9	< 0.001
	*n* (%)	*n* (%)	*n* (%)	
Primiparous^†^	798 (44.9)	625 (43.2)	173 (52.3)	0.002
Smoking (yes)^†^	289 (16.8)	234 (16.2)	55 (16.6)	0.910
*Primary method for IOL*				
Prostaglandin			106 (32.0)	
Balloon catheter			81 (24.5)	
Artificial rupture of membrane			54 (16.3)	
Oxytocin			81 (24.5)	
Failed IOL			11 (3.3)	
Mode of delivery				
Vaginal delivery^†^	1395 (78.5)	1171 (80.9)	224 (67.7)	< 0.001
Vacuum extraction^†^	235 (13.2)	186 (12.9)	49 (14.8)	0.393
Urgent cesarean section	130 (7.3)	77 (5.3)	53 (16.0)	< 0.001
Emergency cesarean section^§^	18 (1.0)	13 (0.9)	5 (1.5)	0.356
Lenght of labor (mean minutes ± SD)^‡^	495 ± 315	507 ± 310	433 ± 332	< 0.001

* *P-*value from Mann–Whitney U-test

^†^ *P-*value from Chi-squared test

^‡^ *P-*value from Wilcoxon rank sum test

^§^ *P-*value from Fisher test

*EPDS* Edinburg postnatal depression scale, *IOL* induction of labor

The women were instructed to assess the occurrence of the sleep disturbances during the past month by the Basic Nordic Sleep Questionnaire (BNSQ) [31] with a five-point scale including questions of ‘general sleep quality’, ‘difficulty to fall asleep/week’, ‘nocturnal awakenings/week and/night’ (two separate questions), ‘awakening too early in the morning (without being able to fall asleep again)/week’, ‘snoring/week’ and ‘witnessed apneas/week’, ‘sleepiness in the morning/week’, ‘daytime sleepiness/week’, and ‘daytime napping/week’ (Appendix 1). In addition, the sum scores of Insomnia (‘difficulty to fall asleep/week’, ‘nocturnal awakenings/week’, and ‘awakening too early in the morning/week’), SDB; ‘snoring/week’ and ‘witnessed apneas/week’), and Sleepiness (‘sleepiness in the morning/week’, ‘daytime sleepiness/week’, and ‘daytime napping/week’) were computed. Furthermore, ‘sleep duration’ (min) and ‘sleep need’ (duration that a woman would like to sleep, min) were assessed, and ‘sleep loss’ was computed by subtracting ‘sleep need’ from ‘sleep duration’ as expressed in minutes.

## Statistical analyses

Based on the power calculation with the sample size of our groups, we are able to detect effect size of 0.17 between groups (at alpha level 0.05 and power 0.8). Power calculation was conducted using the library pwr in R.

The responses for distinct sleep variables were dichotomized: ‘general sleep quality’ (1–3: ‘good sleep quality’ and 4–5: ‘poor sleep quality’); ‘difficulty to fall asleep’, ‘nocturnal awakenings’, and ‘awakening too early in the morning’ (1–3: ‘no disturbance’ and 4–5: ‘yes disturbance’); ‘snoring’ and ‘apneas’ (1–3: ‘occasional snorers/apneas’ and 4–5: ‘habitual snorers/apneas’); and ‘morning sleepiness’, ‘daytime sleepiness’, and ‘napping’ (1–3: ‘no disturbance’ and 4–5: ‘yes disturbance’). Insomnia, SDB, and Sleepiness scores were considered continuous variables. Further, sleep duration (continuous and categorical [< 7 h, 7–9 h, and > 9 h]) and sleep loss (continuous and categorical [< 2 h and ≥ 2 h]) were analysed.

To study the associations between IOL and sleep, the association between each explanatory sleep variable and the dependent variable of IOL was analysed using univariate logistic regression. Then, the analyses were conducted with adjustments for age, BMI, parity, smoking, and EPDS in the early pregnancy. Age, BMI, and EPDS were considered continuous and parity (primiparous/multiparous) and smoking (yes/no), categorical. As the Chi-square test showed a statistically significant association between smoking and education, only smoking was included in the regression model.

As the answering time varied (of all women, 1497 had answered within seven days and five answered later, and in 276, women the time of the reply was unknown), the additional analyses where the sleep disturbances between the two groups (women answering within seven days versus women answering later or with unknown time) were compared using Chi-squared test or Wilcoxon rank sum test when appropriate. In addition, in a post hoc analyses, whether the IOL group had a moderating effect on the association between sleep disturbances and delivery mode (CS versus vaginal delivery) was conducted using logistic regression. Delivery mode was used as the dependent variable. Analyses were adjusted with the same variables as described above.

*P*-values (two-tailed) < 0.05 were considered statistically significant. The 95% confidence intervals (CIs) were calculated for the odds ratios (ORs) and adjusted odds ratios (AORs). All analyses were performed in R (4.0.5, 2021).

## Results

### Clinical characteristics of the study population

Compared to the women in the SOL group, those in the IOL group were more often primiparous, had higher BMI, and more often delivered by CS (Table 1). In all, 9 (2.7%) of the IOL women were induced because of maternal tiredness. From the entire BNSQ data, 0.7–2.0% was missing, except for questions regarding sleep duration, for which 6.5% was missing. The occurrence of sleep disturbances did not differ according to the answering time to the questionnaire (data not shown). In the post hoc analysis, the IOL group had no moderating effect on the association between sleep variables and delivery mode (CS versus vaginal delivery).

### General sleep quality and insomnia symptoms

Table 2 shows the occurrences of sleep disturbances and Table 3 the differences in sleep quality between the groups. There were no differences between the groups in terms of general sleep quality (*P* = 0.186) or Insomnia score (*P* = 0.104). Approximately 40% of the women in both groups had poor general sleep quality. Nearly all women in both groups had nocturnal awakenings almost every night when considered by weekly basis and almost half of the women had nocturnal awakenings at least three times nightly. Difficulty to fall asleep was also frequent, occurring almost in 30% of the women in both groups. Awakening too early in the morning occurred more rarely 21% and 17% of women in the IOL and SOL groups, respectively.

**Table 2:** Occurrences of sleep disturbances in the groups

	Spontaneous labor *n* = 1447		Induction of labor *n* = 331	
	*n* (%)	Mean ± SD	*n* (%)	Mean ± SD
General sleep quality (quite poor or poor)	558 (39.0)		141 (43.0)	
Insomnia score*		9.9 ± 2.1		10.1 ± 2.2
Difficulty to fall asleep/week (yes)	399 (27.8)		93 (28.2)	
Nocturnal awakenings/week (yes)	1358 (94.6)		310 (93.9)	
Nocturnal awakenings/night (yes)	688 (48.1)		144 (43.9)	
Too early morning awakening/week (yes)	240 (16.8)		68 (20.7)	
Sleep Disordered Breathing score^†^		3.0 ± 1.5		3.3 ± 1.7
Snoring/week	241 (17.0)		74 (22.7)	
Witnessed apneas/week (yes)	11 (0.8)		4 (1.2)	
Sleepiness score^‡^		8.6 ± 2.7		8.6 ± 2.6
Morning sleepiness/week (yes)	292 (20.4)		80 (24.3)	
Daytime sleepiness/week (yes)	403 (28.1)		94 (28.7)	
Napping/week	521 (36.6)		113 (34.5)	
Sleep duration (h)		7.8 ± 1.3		7.7 ± 1.4
< 7 h	210 (15.4)		61 (20.2)	
> 9 h	98 (7.2)		25 (8.3)	
Sleep loss^§^ (min)		58 ± 73		64 ± 91
Sleep loss ≥ 2 h	324 (24.5)		77 (25.9)	

Sleep disturbances were adopted from Basic Nordic Sleep Questionnaire (BNSQ) with ratings of 1–5, where 4–5 were considered indicative (‘yes’) of a sleep disturbance

*Insomnia score = Sum score of the answers for ‘Difficulty to fall asleep’, ‘Nocturnal awakenings/week’, and ‘Awakening too early in the morning’

^†^Sleep Disordered Breathing score = Sum score of the answers for ‘Snoring’ and ‘Witnessed apneas’

^‡^Sleepiness score = Sum score of the answers for ‘Sleepiness in the morning’, ‘Daytime sleepiness’, and ‘Napping’

^§^Sleep loss = sleep need–sleep duration

**Table 3:** Associations between sleep disturbances and induction of labor

	Spontaneous labor	Induction of labor			
		OR (95% CI)^*^	*P*-value	AOR (95% CI)^†^	*P*-value
General sleep quality (quite poor/poor)	Ref	1.18 (0.92–1.50)	0.186	1.08 (0.82–1.42)	0.566
Insomnia score^§^	Ref	1.05 (0.99–1.11)	0.104	1.03 (0.96–1.10)	0.406
Difficulty to fall asleep/week (yes)	Ref	1.02 (0.78–1.33)	0.890	1.04 (0.77–1.39)	0.801
Nocturnal awakenings/week (yes)	Ref	0.89 (0.55–1.51)	0.653	0.77 (0.45–1.40)	0.369
Nocturnal awakenings/night (yes)	Ref	0.84 (0.66–1.07)	0.169	0.79 (0.60–1.02)	0.076
Too early morning awakening/week (yes)	Ref	1.29 (0.10–1.74)	0.094	1.08 (0.76–1.50)	0.667
Sleep Disordered Breathing score^││^	Ref	1.09 (1.01–1.17)	**0*.*021**	0.99 (0.91–1.08)	0.835
Snoring/week (yes)	Ref	1.43 (1.06–1.91)	**0*.*017**	1.03 (0.73–1.44)	0.848
Sleepiness score^¶^	Ref	1.01 (0.97–1.06)	0.675	1.00 (0.95–1.05)	0.940
Morning sleepiness/week (yes)	Ref	1.26 (0.94–1.66)	0.115	1.20 (0.87–1.65)	0.258
Daytime sleepiness/week (yes)	Ref	1.03 (0.78–1.34)	0.846	0.93 (0.69–1.25)	0.636
Napping/week (yes)	Ref	0.91 (0.71–1.17)	0.468	0.87 (0.66–1.15)	0.342
Sleep duration	Ref	0.93 (0.85–1.03)	0.163	0.94 (0.85–1.04)	0.219
< 7 h^‡^	Ref	1.41 (1.02–1.94)	**0*.*034**	1.31 (0.91–1.85)	0.133
> 9 h^‡^	Ref	1.24 (0.77–1.94)	0.358	1.15 (0.68–1.87)	0.591
Sleep loss^#^	Ref	1.00 (1.00–1.00)	0.252	1.00 (1.00–1.00)	0.172
≥ 2 h	Ref	1.08 (0.81–1.43)	0.604	1.04 (0.74–1.43)	0.833

Sleep disturbances were adopted from Basic Nordic Sleep Questionnaire (BNSQ) with ratings of 1–5, where 4–5 were considered indicative (‘yes’) of a sleep disturbance

*OR = Univariate analysis (binary logistic regression analysis)

^†^AOR = Adjusted for age, BMI, parity, smoking, and Edinburgh postnatal depression scale (EPDS) in early pregnancy (binary logistic regression analysis)

^‡^ Compared to those who sleep 7–9 h

^§^Insomnia score = Sum score of the answers for ‘Difficulty to fall asleep’, ‘Nocturnal awakenings/week’, and ‘Awakening too early in the morning’

^││^Sleep Disordered Breathing score = Sum score of the answers for ‘Snoring’ and ‘Witnessed apneas’

^¶^Sleepiness score = Sum score of the answers for ‘Sleepiness in the morning’, ‘Daytime sleepiness’, and ‘Napping’

Bolded *P*-values show statistical significance level < 0.05

### Sleep disordered breathing

In the IOL and SOL groups, 23% and 17% of women, respectively, were habitual snorers (Table 2). Thus, women in the IOL group were more likely to be habitual snorers (*P* = 0.017); however, the difference lost the statistical significance in adjusted analysis (*P* = 0.848) (Table 3). Witnessed apneas were rare in both groups (Table 2).

### Sleepiness symptoms

Sleepiness scores were similar between the groups (*P* = 0.675) (Table 3). Among distinct sleepiness symptoms, napping was the most frequent in both groups (35%) (Table 2). Furthermore, daytime sleepiness was common in both groups (28%) (Table 2). Although morning sleepiness was less common, it occurred also quite frequently (IOL 24% and SOL 20%, respectively) (Table 2).

### Sleep duration and sleep loss

The mean sleep duration was similar in both groups (*P* = 0.163) (Table 3). Nevertheless, when sleep duration was considered categorical, women in the IOL group were more likely to be short sleepers compared to those in the SOL group (*P* = 0.034) (Table 3). However, this finding lost statistical significance in the adjusted model (*P* = 0.133) (Table 3). In sleep loss, the two groups showed no differences (*P* = 0.252) (Table 3).

## Discussion

Our study was one of the first to evaluate the associations between sleep disturbances and IOL. Only minor findings emerged: habitual snorers and short sleepers were more likely to have IOL. However, these associations vanished in the adjusted models, indicating that sleep disturbances per se do not have a strong impact.

Previous literature evaluating sleep quality in late pregnancy is quite unanimous that sleep disturbances are frequent [7, 11, 12, 14, 15]. Using the BNSQ, in our two previous studies, in a pilot study with 78 women [11] and a follow-up study with 1858 women [12], 15% and 30% women, respectively, reported poor sleep quality in late pregnancy. Another Finnish study evaluating sleep also with BNSQ in 325 women, reported that 30% of late pregnant women had restless sleep [15]. Further, according to a meta-analysis including 42 studies and using the Pittsburgh Sleep Quality Index questionnaire for sleep quality evaluation, in perinatal period the prevalence of poor sleep was 45% [32]. Furthermore, in a study of 2427 pregnant women also using the Pittsburgh Sleep Quality Index questionnaire, 76% of the women experienced poor sleep quality across all months of pregnancy [14].

Despite of the previously described deterioration in sleep quality during pregnancy, only one earlier study has assessed the relationship between sleep and IOL. Measuring sleep with a wrist actigraphy, Beebe and Lee [33] enrolled 35 women and found no differences between IOL and SOL groups in sleep duration and nightly wake-time after sleep onset five days before labor [7]. Though, interestingly, therapeutical rest, usually implemented by using pain relief medication in early labor, have been shown to decrease the risk of getting IOL. Furthermore, maternal tiredness is important indication for IOL; according to the study by Dögl et al. [6], about one third of elective IOLs were performed because of mothers request, and indication in every fifth of these IOLs was maternal fatigue and tiredness.

In our study, SDB, especially snoring, as well as short sleep were associated with the likelihood of IOL; however, the associations vanished after adjustment. We adjusted models with age and obesity, which are independent risk factors for SDB development [34], and with comorbidities, which may cause IOL [19, 35, 36]. As for sleep duration, Lee et al. [23] showed that short sleep, sleep under 6 h, was a risk factor for CS. This association was also shown in a study including only IOL women [37]. In addition, short sleep is associated with gestational diabetes [36], which increases the risk of IOL. Overall, although sleep has some associations with IOL, the likelihood of induction seems to be directly associated with factors other than sleep. Nevertheless, taken all these interactions together, sleep may influence as a mediator in labor and in IOL and thus, should not be ignored.

The strengths of our study are the large size of the study population and the use of BNSQ, a widely used sleep questionnaire among pregnant women [11, 12]. However, BNSQ was filled in after delivery in postpartum ward, and even though women were instructed to assess their sleep during the past month, which also covered pre-delivery time, the answers could have been influenced by the entire labor process and postpartum time. Therefore, pre-delivery evaluation of the occurrence of sleep disturbances could have been interesting and warrants more research in future. Furthermore, sleep was assessed only in late pregnancy, and therefore, our results cannot necessarily be extrapolated to women with long-term sleep disturbances. No sleep architecture was measured, which could have given another perspective to sleep quality. The women in our study had mainly uncomplicated full-term pregnancies, and therefore, our results cannot be generalized to women with preterm delivery and pregnancy complications. The study population was relatively healthy, but the medical information was partly based on the self-report of the women, and thus, all pre-pregnancy or gestational comorbidities, which might have influenced on either sleep or IOL, could not be excluded.

Sleep disturbances are common, especially in late pregnancy, and therefore, they may interfere with labor. Although in our study sleep disturbances, especially insomnia symptoms, occurred frequently, they were not associated with the likelihood of ending up to IOL. Still, part of the women in our study had IOL because of maternal tiredness, given that sleep disturbances and plausible daytime consequences, like sleepiness and tiredness, cannot totally be ignored in women in labor. As the rates of IOL are increasing, more research for risk factors for IOL are warranted.

## Data Availability

The data that support the findings of this study are available from the corresponding author, (HL), upon reasonable request.

## Appendix

### Supporting information: The Basic Nordic Sleep Questionnaire (BNSQ)

1. Have you had difficulties to fall asleep during the past month?
  1. never or less than once per month
  2. less than once per week
  3. on 1–2 nights per week
  4. on 3–5 nights per week
  5. every night or almost every night
2. **How often have you woken up at night during the past month?**
  1. never or less than once per month
  2. less than once per week
  3. on 1–2 nights per week
  4. on 3–5 nights per week
  5. every night or almost every night per week
3. **How many times do you usually wake up during a night (during the past month)?**
  1. I do not wake up at night usually
  2. once per night
  3. twice per night
  4. 3–4 times per night
  5. at least 5 times per night
4. **How often have you awakened too early in the morning without being able to fall asleep again during the past month?**
  1. never or less than once per month
  2. less than once per week
  3. on 1–2 days per week
  4. on 3–5 days per week
  5. every night or almost every night
5. **How well have you been sleeping during the past month? (general sleep quality)**
  1. well
  2. quite well
  3. neither well nor poorly
  4. quite poor
  5. poor
6. **Do you snore while sleeping?** (ask other people if you are not sure)
  1. never or less than once per month
  2. less than once per week
  3. on 1–2 nights per week
  4. on 3–5 nights per week
  5. every night or almost every night
7. **Have you had breathing pauses (sleep apneas) while sleeping?** (ask other people if you are not sure)
  1. never or less than once per month
  2. less than once per week
  3. on 1–2 nights per week
  4. on 3–5 nights per week
  5. every night or almost every night
8. **Do you feel excessively sleepy in the morning after awakening during?**
  1. never or less than once per month
  2. less than once per week
  3. on 1–2 days per week
  4. on 3–5 days per week
  5. every day or almost every day
9. **Do you feel excessively sleepy during the daytime?**
  1. never or less than once per month
  2. less than once per week
  3. on 1–2 days per week
  4. on 3–5 days per week
  5. every day or almost every day
10. **How often do you take naps during the day?**
  1. never or less than once per month
  2. less than once per week
  3. on 1–2 days per week
  4. on 3–5 days per week
  5. every day or almost every day
11. **How long do you usually sleep per night? ___hours ___minutes**
12. **How many hours of sleep do you need per night (how many hours would you like to sleep if you had possibility to sleep as long as you need to)? ___hours ___minutes**

## Abbreviations

AOR: Adjusted odds ratio
BMI: Body mass index
BNSQ: Basic Nordic Sleep Questionnaire
CI: Confidence intervals
CS: Cesarean section
EPDS: Edinburgh Postnatal Depression Scale
IOL: Induction of labor
SDB: Sleep disordered breathing
SOL: Spontaneous onset of labor
Th: T helper lymphocyte
